# A rare content of congenital inguinal hernia: a case report of splenogonadal fusion

**DOI:** 10.1186/s12887-019-1807-x

**Published:** 2019-11-10

**Authors:** Xi Xiang, Yong Jiang, Ju-xian Liu, Li Qiu

**Affiliations:** 1 Department of Medical Ultrasound, West China Hospital, Sichuan University, No.37 Guo Xue Xiang, Chengdu, 610041 Sichuan Province China; 2 Department of Pathology, West China Hospital, Sichuan University, Chengdu, Sichuan China

**Keywords:** Congenital malformation, Inguinal hernia, Pathology, Splenogonadal fusion, Ultrasound

## Abstract

**Background:**

Splenogonadal fusion (SGF) is a rare congenital malformation that occurs during embryonic development. SGF typically presents as a left-sided scrotal swelling, left inguinal hernia, scrotal mass, or cryptorchidism. Therefore, it is easily misdiagnosed, and unnecessary orchiectomy may occur. This study aimed to report a rare case of SGF.

**Case presentation:**

A 5-month-old male child presented with a history of obvious left scrotal swelling for 1 month, which progressively worsened 10 h before the hospital visit. The ultrasound examination exhibited a solid mass in the left scrotum, with echo quite similar to that in the testicle and went up into the abdominal cavity through an identical echogenic band structure. After surgical resection, the pathological examination confirmed that the submitted tissue was spleen tissue with extensive bleeding.

**Conclusion:**

Ultrasound can provide important information regarding the diagnosis of SGF. The possibility of SGF should be considered for further differential diagnosis in the case of similar patients.

## Background

Splenogonadal fusion (SGF) is a rare congenital malformation in which the spleen is abnormally connected to the gonads or rarely to the mesonephric structures such as vas or epididymis [1]. SGF typically presents as a left-sided scrotal swelling, left inguinal hernia, scrotal mass, or cryptorchidism [2]. To date, only a few cases of SGF have been reported worldwide. However, ultrasound manifestations of SGF are seldom reported. As a nonradiative and economical inspection method, ultrasound is often used as the first choice for superficial mass inspection, including scrotal lesions. SGF is usually misdiagnosed in most cases as testicular tumors, necrotic intestines, multiple testis, and so on. A priori knowledge of this condition can avoid unnecessary orchiectomy.

## Case presentation

A 5-month-old male child with a history of obvious left scrotal swelling for 1 month was admitted to the emergency department of West China Hospital of Sichuan University (Chengdu, China). In the beginning, swelling and regression of the left scrotum alternated. The parents did not pay enough attention. However, 10 h before the visit, the swelling of the left scrotum progressively worsened and the color of the left scrotum wall turned red. No other abnormalities were found in the physical examination except for a soft and nonreturnable mass in the left scrotum. Specifically, the transillumination of the scrotum was negative.

**Fig. 1:**
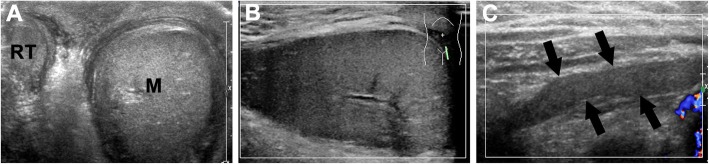
Ultrasound manifestation: **a** A mass (M) in the left scrotum with echo similar to that in the right testis (RT). **b** No blood flow signal in the mass. **c** The connection band (arrows). One end of the band was connected to the left scrotum mass, and the other end was extended to the abdominal cavity

The patient immediately underwent an ultrasound examination of the scrotum under the guidance of pediatric surgeons. Gray-scale ultrasound revealed a solid mass (6 × 4 × 3 cm^3^) in the left scrotum (Fig. 1a and b), with echo quite similar to that in the testicle and went up into the abdominal cavity through an identical echogenic band structure (Fig. 1c). Due to the obstruction of the intestinal gas, part of the structure in the abdominal cavity could not be displayed. The left testicle was pushed to the bottom of the scrotum by the solid mass, and the size of the left testicle (10 × 5 × 6 mm^3^) was slightly smaller than that of the right one (13 × 7 × 8 mm^3^). Moreover, color Doppler ultrasound showed that the blood flow signal was more abundant in the left testicle than in the right one. Confusingly, no blood flow signal was found inside the mass in the left scrotum presented (Fig. 1b). The ultrasound examination results suggested the left incarcerated inguinal hernia with possible inner necrosis.

**Fig. 2:**
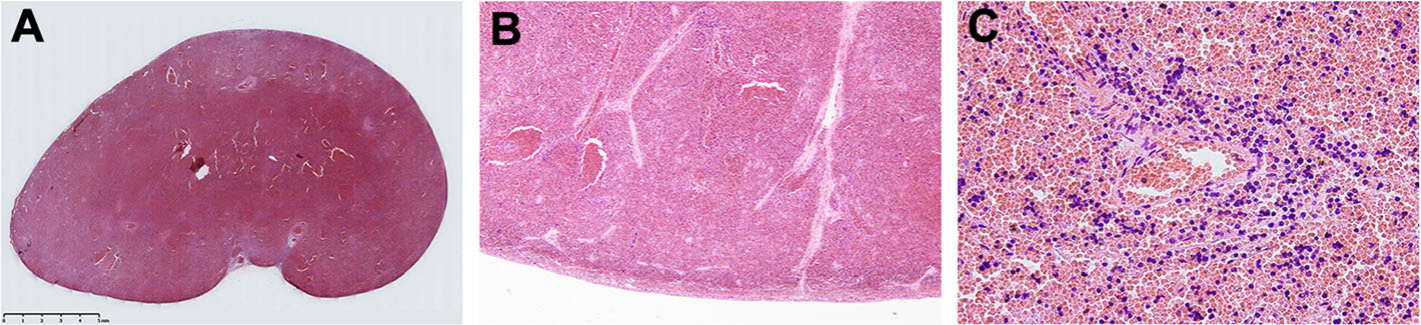
Pathological manifestation: **a** Slice under the naked eye. **b** Pathological findings suggested spleen tissue with extensive bleeding (× 40). **c** Pathological findings suggested spleen tissue with extensive bleeding (× 400)

The patient underwent emergency surgical exploration considering the possible necrosis. The mass appeared dark red during the surgery. One end of the mass was connected to the left epididymis, and the other end was connected to the abdominal cavity through the inner ring orifice. The laparoscopic exploration revealed a duct-like structure beneath the spleen of the left upper abdomen connected to the mass in the scrotum. As the mass was highly likely to be necrotic and simultaneously affected the repair of the left inguinal hernia, the left scrotum mass was removed completely with the consent of the patient’s parent. The intact left testis was preserved, while the epididymis was retained as much as possible so as to not affect the function of the epididymis. The pathological results showed that the submitted tissue was spleen tissue with extensive bleeding (Fig. 2). The patient recovered quickly after the surgery and was followed up using ultrasound after 1, 2, and 4 months of the surgical procedure. No more obvious abnormality was found in the scrotum.

## Discussion and conclusion

Inguinal hernia repair is one of the most common surgical procedures performed in children worldwide [3, 4]. Inguinal hernia is a relatively common surgical problem in children, with a reported incidence ranging from 0.5 to 4.5% [5, 6]. The cumulative incidence of inguinal hernia repair is 7% in male patients and 1% in female patients for children aged less than 15 years [7]. Splenogonadal fusion is a rather specific type of inguinal hernia in a male patient, characterized by its ectopic spleen tissue. As a rare congenital malformation, SGF is associated with other malformations such as cleft palate, micrognathia, cardiac anomalies, spinal anomalies, and so on [8].

Classically, splenogonadal fusion is classified into a continuous type that depicts a connection between the spleen and the gonad, and a discontinuous type that may demonstrate an ectopic focus of splenic tissue attached to the gonad [9]. These two types occur at relatively equal frequencies [10]. In the continuous type, the connection band may provide strong evidence for the diagnosis of SGF. The connection between the mass in the scrotum and the spleen can be determined by the connection band. However, the discontinuous type may have a higher rate of misdiagnosis compared with the continuous one. For example, it can be misdiagnosed as testicular or epididymal tumors, thus leading to inappropriate surgical decisions. The patient in this study belonged to the continuous type. Regrettably, this study failed to show the complete connection band between the mass and the spleen because of the obstruction of gas in the intestine. Finally, the lesion was completely removed during the surgery. No abnormalities occurred during the follow-up. The parents of the child were satisfied with the entire diagnosis and treatment process.

Generally considered, the adherence of the splenic primordium to structures derived from the mesonephric ridge occurs between the fifth and eighth weeks of gestation. The descent of the testis seems to draw out the developing spleen into a long band in some cases or to detach a portion of the splenic primordium and carry it down with the descending testis in others [11]. Abnormal development at this stage can lead to SGF.

Magnetic resonance imaging is valuable for the diagnosis of SGF [10]. However, it is less suitable for infants and young children who are not easily controlled unless sedatives are used. SGF is mostly found in infants and young children. In the early years, 99Tc-sulfur colloid imaging was used to identify ectopic splenic tissue [12]. However, the role of ultrasound in the diagnosis of SGF has rarely been reported. This study depicted the performance of ultrasound in the diagnosis of SGF. The gray-scale ultrasound revealed a mass in the left scrotum with echo similar to that in the testicle, and the echogenic band structure went up into the abdominal cavity; however, this might not be the case in different types of SGF. The ultrasound has its own limitations and is susceptible to abdominal gas. Under conditions of fasting, the ultrasound may exhibit that the band is connected to the spleen in the continuous type by switching probes of different spectra. The blood supply is decided based on the necrosis in the inside of the mass.

In an early study, 37% of the reported cases were associated with orchiectomy [13], and unnecessary orchiectomy led to a high rate of misdiagnosis. Clinical awareness of SGF may avoid more invasive surgery and even unnecessary orchiectomy.

SGF is a rare congenital malformation that typically presents as a left-sided scrotal swelling, inguinal hernia, scrotal mass, or cryptorchidism. Ultrasound can provide important information regarding the diagnosis of SGF with seldom contraindications, no radiation, and no need for sedatives. Hence, it is more practical for infants and young children. The possibility of SGF should be considered for further differential diagnosis in the case of similar patients.

## Data Availability

All data that supporting these findings of this article are included with the manuscript.

## Abbreviation

SGF: Spleno-gonadal fusion
